# Putting 3D modelling and 3D printing into practice: virtual surgery and preoperative planning to reconstruct complex post-traumatic skeletal deformities and defects

**DOI:** 10.1051/sicotj/2016043

**Published:** 2017-02-21

**Authors:** Kevin Tetsworth, Steve Block, Vaida Glatt

**Affiliations:** 1 Department of Orthopaedic Surgery, Royal Brisbane Hospital Herston Queensland 4029 Australia; 2 Orthopaedic Research Centre of Australia Herston Queensland 4029 Australia; 3 4WEB Medical Frisco TX 75033 USA; 4 Department of Orthopaedic Surgery, University of Texas Health Science Center San Antonio TX 78229 USA; 5 Institute of Health and Biomedical Innovation, Queensland University of Technology Brisbane Queensland 4059 Australia

**Keywords:** 3D printing and modelling, Orthopaedics, Virtual surgery planning, Limb salvage, Printing, three-dimensional

## Abstract

3D printing technology has revolutionized and gradually transformed manufacturing across a broad spectrum of industries, including healthcare. Nowhere is this more apparent than in orthopaedics with many surgeons already incorporating aspects of 3D modelling and virtual procedures into their routine clinical practice. As a more extreme application, patient-specific 3D printed titanium truss cages represent a novel approach for managing the challenge of segmental bone defects. This review illustrates the potential indications of this innovative technique using 3D printed titanium truss cages in conjunction with the Masquelet technique. These implants are custom designed during a virtual surgical planning session with the combined input of an orthopaedic surgeon, an orthopaedic engineering professional and a biomedical design engineer. The ability to 3D model an identical replica of the original intact bone in a virtual procedure is of vital importance when attempting to precisely reconstruct normal anatomy during the actual procedure. Additionally, other important factors must be considered during the planning procedure, such as the three-dimensional configuration of the implant. Meticulous design is necessary to allow for successful implantation through the planned surgical exposure, while being aware of the constraints imposed by local anatomy and prior implants. This review will attempt to synthesize the current state of the art as well as discuss our personal experience using this promising technique. It will address implant design considerations including the mechanical, anatomical and functional aspects unique to each case.

## Introduction

There is currently a tremendous level of interest in developing uses for 3D modelling and 3D printing in orthopaedic surgery, as demonstrated by a number of recent publications [1–6]. Since the advent of 3D printing, the technology has revolutionized and gradually transformed manufacturing across a broad spectrum of industries, and healthcare is no exception. Its popularity in medicine and surgery has grown rapidly over the past several years, and new applications are evolving at an accelerated pace. 3D printing describes any of the various techniques used for making physical objects from graphical computer data through an additive process, laying down successive layers of material under computer control using a variety of metals or plastics [2]. This revolutionary technology has now become far more accessible and affordable, and is already mainstream in many areas of medicine [6].

Nowhere is this more apparent than in orthopaedics, and many surgeons already incorporate aspects of 3D modelling and virtual procedures into their routine clinical practice [6]. It is easy to overlook how truly pervasive 3D modelling and 3D printing have become in contemporary orthopaedic surgery. Many implants are now developed and designed based on 3D models of pertinent regional anatomy [7–9]. One manufacturer now provides custom arthroplasty components, tailored to the subtle nuances of each patient’s specific anatomy [10]. More importantly, hundreds of procedures are conducted daily where 3D modelling and virtual surgery have become an integral part of the actual procedure. In fact, 3D modelling and 3D printing have become standard practice in some operating theatres, although most often virtual surgical procedures are used for preoperative planning [11–17] or intra-operatively through navigation [18–20]. It is so prevalent that these technologies now play an important role in most of the complex orthopaedic cases currently treated around the world. This is most evident in the realm of adult reconstructive surgery [21, 22] and joint arthroplasty [8, 9, 12, 20, 23–31], but it is rapidly permeating every aspect of orthopaedic surgery, including trauma [7, 32–41], spine [42–45], hand [39, 40, 46–49], shoulder [14–17, 50, 51], tumour [52–54] and sports medicine [50].

The possibility of customized manufacturing using 3D printers has opened new horizons within complex post-traumatic limb reconstruction. One such novel strategy involves patient-specific custom 3D printed titanium truss cages that can be used to address the extremely difficult problem of segmental bone loss often associated with post-traumatic deformities. Truss configurations are well known in engineering and provide the most strength with the least mass. These constructs are mechanically robust, with structural stability that facilitates immediate motion and early weight bearing. This unique configuration also acts as a lattice for bone graft and can be used in combination with the Masquelet induced membrane technique [55–60]. These implants are designed in a virtual surgical procedure that provides patient-specific options for re-alignment and restoring length. The staged approach plays a critical role in the success of the procedure, and the membrane develops while the custom implant is first designed and then produced. The induced membrane creates a more favourable recipient bed for the reconstruction, producing growth factors that encourage more rapid incorporation of the graft [59, 60].

The primary focus of this review is to describe the most important elements to consider when performing virtual surgery as a planning procedure to design patient-specific 3D printed implants, highlighting certain aspects that may be unique to specific conditions. This discussion will address virtual surgical planning and implant design considerations, indicating how this process could be successfully introduced into clinical practice.

## Methods: treatment protocol and rationale

**Figure 1.**
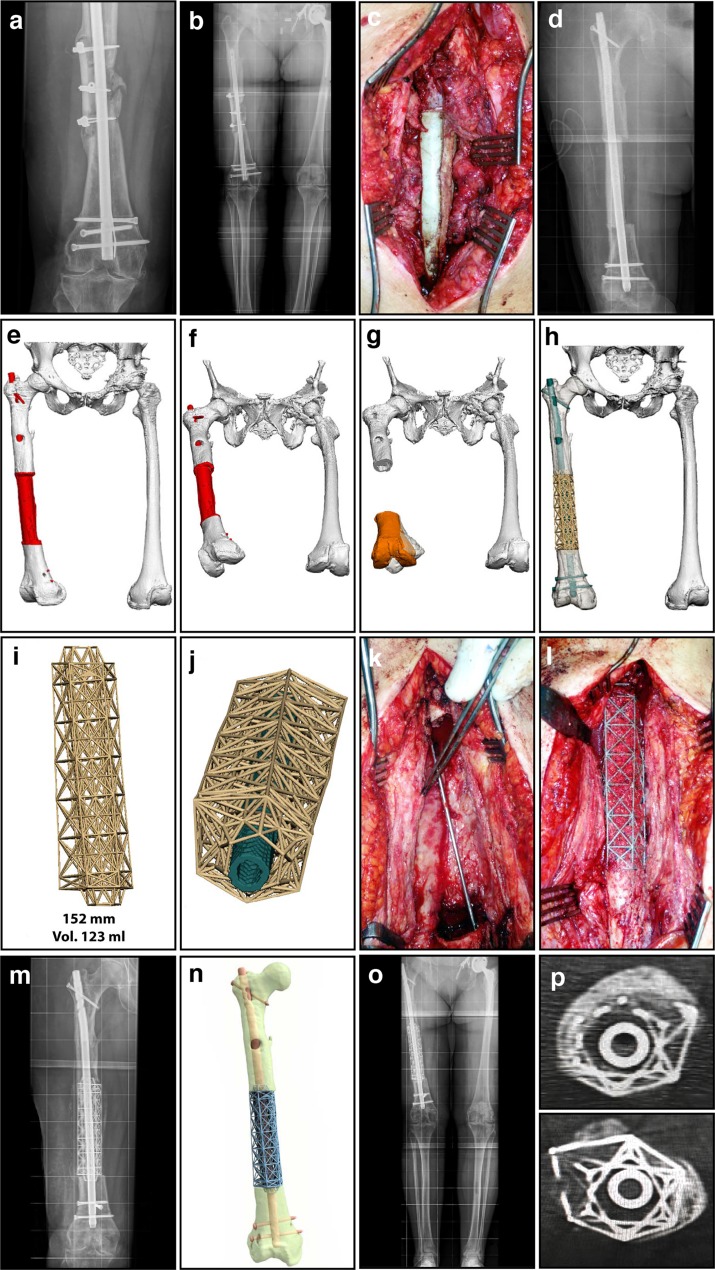
Diaphyseal femoral segmental defect (15.2 cm) – infected non-union. (a) Anteroposterior (AP) radiograph of femoral mid-diaphyseal infected non-union, with a sequestrum consisting of the remnants of a prior intercalary allograft. (b) Long-standing radiograph of femoral mid-diaphyseal infected non-union, demonstrating alignment and limb lengths. (c) Intra-operative image during the 1st stage, illustrating a temporary antibiotic-loaded PMMA spacer. (d) Radiograph showing an antibiotic-loaded PMMA spacer fashioned to completely fill the defect, enveloping the bone at both the proximal and distal ends. (e) 3D modelling image showing the antibiotic spacer spanning the defect. (f) Axial view of 3D modelling image, demonstrating a 32° internal rotation deformity. (g) 3D virtual procedure images showing the distal fragment internal rotation of 32°, malaligned in 5° excess valgus, and flexed 9°, with a 12 mm residual limb length discrepancy. The planning for correcting the orientation of the distal fragment (amber), restoring its normal anatomic position by using the mirrored image of the contralateral uninvolved normal limb as a template. (h) 3D virtual procedure images showing the truss cage implant, designed to allow stabilization by a large diameter nail. (i) Final titanium truss implant design for the mid-diaphyseal femur, with tapered intramedullary extensions to improve torsional and translational stability. (j) The truss implant design here incorporates an axial hole designed to fit a suitable IM nail for stabilization of the mid-diaphyseal femur. (k) Intra-operative image during the second stage after the PMMA spacer was removed, demonstrating the membrane. (l) Intra-operative image during the second stage, following insertion of the titanium implant with bone graft packed into the open cells of the truss cage. (m) AP radiograph illustrating the final position of the implant, with a nail inserted through the truss cage locked proximally and distally. (n) Computer rendered image of the definitive reconstruction, stabilized with a nail inserted through the truss cage. (o) Long-standing radiograph of the lower extremities post-operatively, demonstrating excellent alignment and equal limb lengths. (p) CT scan at six-month post-operative confirming solid incorporation of bone graft, best demonstrated at the host/implant junctions proximally and distally.

**Figure 2.**
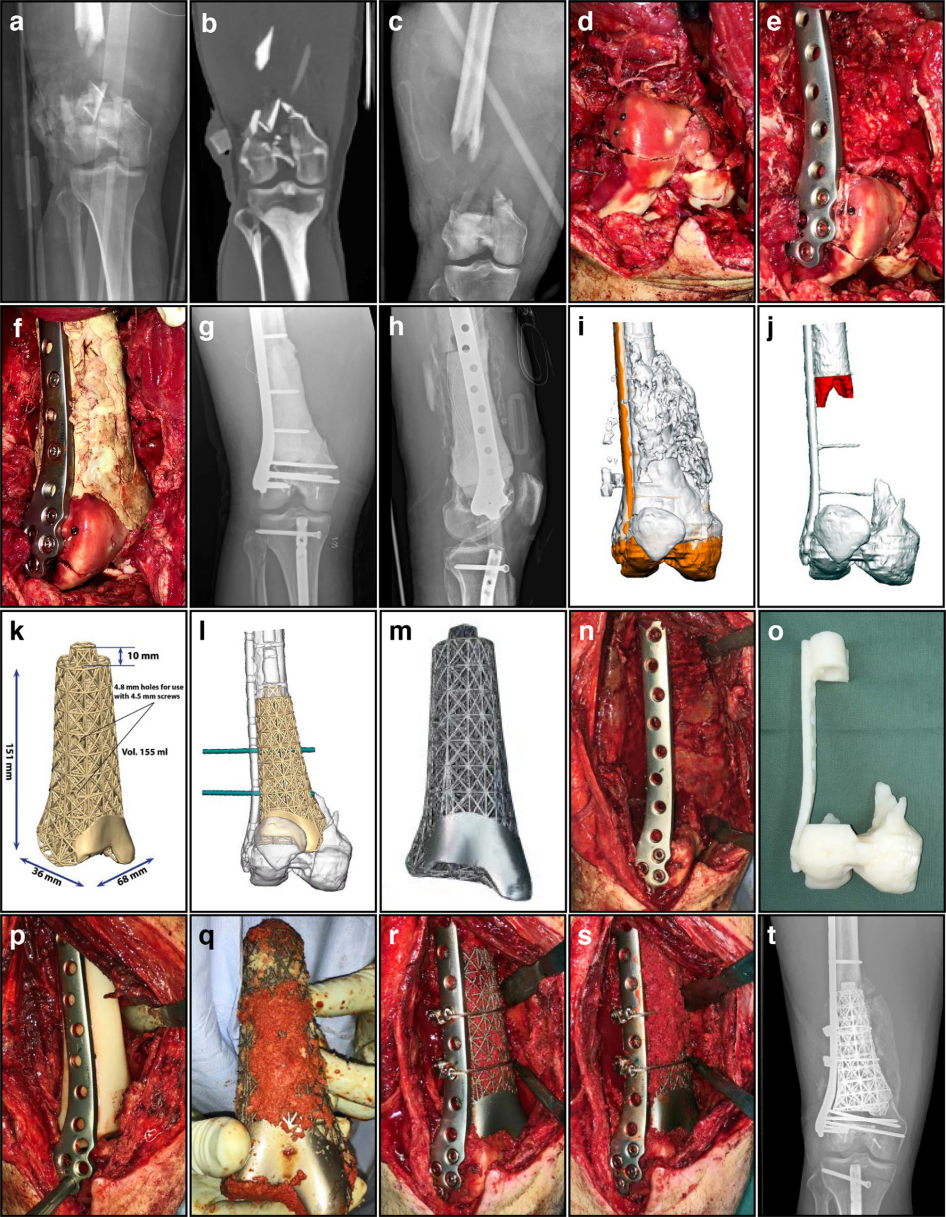
Metadiaphyseal distal femoral segmental defect (15.1 cm) – Grade 3A open fracture. (a) AP radiograph of this Grade 3A open R distal femur fracture immediately following the injury. (b) Initial CT scan demonstrating loss of bone stock and cartilage centrally, with less than 6 mm of bone adjacent to the intercondylar notch. (c) AP radiograph of the distal femur after initial debridement and spanning external fixation. (d) Intra-operative photos during the 1st stage, with reconstruction of the highly comminuted intra-articular extension of the fracture. (e) Intra-operative image showing the fracture reduced anatomically and then stabilized with a locked plate. (f) Intra-operative image during the 1st stage showing the PMMA spacer temporarily filling the defect, augmenting mechanical support of the distal segment while also providing a surface for the development of an induced membrane. (g) AP radiograph following stabilization with a lateral locked plate, incorporating an antibiotic-loaded PMMA spacer. (h) Lateral radiograph of the distal femur with plate and temporary PMMA spacer. (i) The patient-specific 3D printed titanium truss implant was produced after first conducting a virtual surgical procedure. (j) Additional bone was resected (red) to facilitate the actual procedure and to maximize contact and inherent stability. (k) Dimensions of the 3D printed titanium truss implant, designed with a tapered intramedullary extension proximally that increases stability significantly. (l) The truss implant design included trajectories for screw holes that correspond to the existing implant (green). (m) The final implant was produced with a polished surface to articulate with the patella, indicating extent of cartilage loss. The design of the implant very closely mimics the contours and dimensions of the original bone. (n) Intra-operative image during the second stage, with carefully opened and preserved membrane after the PMMA spacer was removed. (o) 3D printed acrylic model of the anticipated final skeletal defect, used to confirm satisfactory fit and alignment of the implant. (p) Intra-operative image showing insertion of a plastic template to assess the adequacy of the final resection, used as a trial before inserting the implant. (q) Before implantation, the truss construct was filled with autologous and allogeneic cancellous bone graft mixed with powdered vancomycin, then manually packed into the open cells of the truss implant. (r) Intra-operative image showing the truss implant filled with bone graft and inserted into the defect, with additional fixation including two cerclage cables. (s) Intra-operative image during the second stage, showing additional bone graft packed over the anterior and medial aspects of the truss implant after definitive fixation was completed. (t) AP radiograph demonstrating early incorporation of the bone graft, with a solid column of dense bridging bone visible medially four months after the procedure.

Using patient-specific custom 3D printed titanium truss cages, in conjunction with the Masquelet technique, is a novel strategy that fully exploits the benefits of virtual surgery as a planning procedure. Based on our limited experience, and the few other reports in the literature [35, 36], this treatment is recommended for post-traumatic segmental bone defects in adults, generally exceeding 8 cm in length. The distal femoral juxta-articular (meta-diaphyseal) region is preferred, particularly when the remaining distal bone is less than 2 cm in length. These implants should be used in compromised hosts, where spontaneous bone growth is less likely to successfully bridge the gap (Figures 1 and 2).

The first stage requires aggressive debridement, preliminary stabilization and use of the Masquelet technique incorporating antibiotic-loaded polymethylmethacrylate (PMMA) as a temporary spacer [55–60]. This spacer should be configured to very closely mimic the size and shape of the original bone that is to be reconstructed (Figures 1c and 2f–2h), following the principle of “replace like with like”. The temporary construct must be rigid enough to facilitate early mobilization and allow unrestricted range of motion (ROM).

The purpose of this temporary antibiotic PMMA spacer is to deliver high concentrations of local antibiotic while simultaneously preserving space for the definitive osseous reconstruction, to greatly enhance mechanical stability and to create an induced membrane that has biological activity and encourages bone graft incorporation by containing the graft and providing a more receptive recipient bed. The PMMA spacer should remain in the defect approximately 8–12 weeks. While the membrane develops, this intervening time period is used for virtual surgery and planning for secondary reconstruction, including design of a truss cage suitable for a given anatomic location. Consideration must be given to the type of skeletal stabilization that will be employed in the definitive construct (intramedullary (IM) nail or internal fixation using a plate and screws).

The second stage follows an interval of approximately 8–12 weeks, to allow the membrane to adequately develop. Of course, this will depend on the nature of the injury, the severity of the soft tissue wounds, the quality of the host and their physiologic reserve. At this point the defect site is ready for definitive reconstruction with a custom truss cage placed into the Masquelet induced membrane (Figures 1k and 2n). The reconstruction must include adequate bone graft/substitute volume to fill the defect and bridge the void between intact host bone, both proximal and distal (Figures 1l and 2q–2s). The second stage is followed by physiotherapy including immediate unrestricted ROM exercises, and progression to full weight bearing (FWB) over 4–6 weeks. Routine clinical review is conducted at intervals with plain radiographs (Figures 1m, 1o and 2t), and a computed tomography (CT) scan at six months to assess graft incorporation and to confirm restoration of skeletal continuity (Figure 1p).

The surgeon must recognize the importance of geometry in three dimensions and should be aware of spatial limitations as the implant is inserted into the skeletal defect. Moreover, if the original fixation is left completely intact it is the most highly constrained condition, and the implant design will be extremely critical to the success or failure of this tactic. If the fixation is to be slightly modified (for example by screw removal), the original construct can be considered less constrained. Although changing the original fixation completely is sometimes an attractive option, there is a trade-off between the freedom gained and the potential complete loss of orientation. Once the initial implants are removed, it can be extremely difficult to maintain the precise position of the bony fragments in space with respect to one another. Because the virtual procedure was completed using certain specific fixed landmarks, if the initial orientation is lost it can be very difficult to confirm whether the implant has actually restored the anatomical relationship of the skeletal elements completely to normal.

Patient-specific 3D printed titanium truss cages can bridge these defects, but must be filled with bone graft to definitively complete the reconstruction. A central phantom can be generated to take the place of the IM nail as the implant is loaded with bone graft, as this space will ultimately be occupied by the nail and therefore does not need to be filled. The volume of the implant depends on its size and shape; the “open volume” of the implant excludes the volume of the struts themselves, the “strut volume”. The open volume therefore represents the volume required to completely eliminate any air within the space occupied by the implant. For large segmental defects this is an extremely important consideration, and it is necessary to obtain adequate bone graft/substitute volume when planning definitive surgery as a second stage.

## Virtual surgery and planning for secondary reconstruction

Virtual surgery planning is best described as an online collaboration between the surgeon, the orthopaedic engineering professional and the biomedical design engineer. During this planning session, the patient’s bony anatomy is studied and manipulated to restore to normal before discussing the implant solution and method of fixation. The surgeon explains the indication and the expected approach, and instructs the engineers on the desired surgical technique. Some surgeons prefer to look through the individual slices of the CT scan, suggesting they have less confidence in the reliability and accuracy of the 3D model; other surgeons have explicit trust in the fidelity of the 3D model. It is often useful to have some transparency in the model to be able to fully assess certain aspects of the pathoanatomy during the planning session.

It is critically important to appreciate the differences between a virtual procedure and an actual procedure. In the virtual procedure an osteotomy or resection can be performed repeatedly, as many times as are necessary to achieve perfection. In the actual procedure you are limited to a few attempts at most. In the virtual procedure there are no anatomical constraints, and the region of interest can be exposed completely. In the actual procedure the anatomy is a constant issue, and adequate exposure and visualization may at times be difficult to achieve. The dissection must respect pertinent anatomy and preserve local vasculature. The physiology of the host is also an important consideration, and blood loss must be limited as much as possible. Time under anaesthesia is also a factor, both with respect to controlling blood loss as well as limiting fatigue and maintaining the concentration of the surgeon.

The time invested in virtual surgical planning procedures will almost always pay dividends on the day of the actual procedure. It is important for the surgeon to be actively involved in the process, to understand and appreciate what can be accomplished intra-operatively. Only the surgeon will have knowledge of the specifics of the planned procedure, and these must be expressed so that the orthopaedic and design engineers are able to take this into consideration. Being actively engaged in the virtual planning will result in the surgeon becoming more familiar with the procedure and better prepared. This will inevitably translate into surgeon confidence that should then be reflected in the technical performance of the procedure itself.

At the start of the virtual planning session, the surgeon and the design engineers must have a common understanding of the intended goals of the reconstruction with a realistic expectation of the anticipated outcome, and be aware of the limits imposed by the severity of the pathology. The planning process then begins with a review of the pertinent history of the patient, their general health, and the particulars of the mechanism of injury, focusing on the specifics of the most recent procedure, including the details of any distorted or destroyed anatomy (Figures 1 and 2).

The intricacy of this process demands close interaction between a given orthopaedic engineering professional, design engineer and specific surgeon. The design engineer will be largely unaware of the limitations and constraints of anatomy, and will not necessarily take this into consideration. They may also be unfamiliar with many of the standard medical terms, such as varus/valgus, or anteversion. The orthopaedic engineer serves as a project manager and coordinates the interests of the surgeon with the talents of the design engineer driving the software. The surgeon, orthopaedic engineer and design engineer must collectively discuss the patients’ anatomy, specifically the pathoanatomy.

Before planning on how to reconstruct the affected region the participants must agree on repositioning the segments of the 3D rendered bone model, to restore normal anatomy. The orientation of the skeletal elements both proximal and distal must first be returned to a normal anatomical relationship with one another, and after this corrected position has been attained, it must then be maintained while the implant is inserted and definitive stabilization is achieved. In a virtual procedure this is often most easily accomplished by first modelling the unaffected contralateral limb, when available. This can then be “mirrored” from the opposite side and serves as an ideal template to most closely mimic the original size, shape and contours of the affected bone (Figures 1e–1h) [48, 49]. Even when there is a bone defect it is important to first reposition the remaining intact skeletal elements, both proximal and distal, to restore them to their original orientation with respect to each other. This must take into account angular deformities (varus/valgus, flexion/extension and oblique plane), rotational abnormalities and axial disorders (length).

It is also critical to determine what additional bone may need to be resected to obtain a stable platform, one that provides inherent mechanical stability. This involves the proximal bone/implant interface, the distal bone/implant interface, as well as the stability of the entire bone/implant composite. This generally requires flat, smooth bone surfaces, perpendicular to the mechanical axis or load that is anticipated. Ideally, in a long bone, this will require two parallel surfaces that are perpendicular to the mechanical axis. Once completed, one can begin to model the implant with the goal of recreating the normal anatomy of the affected skeletal segment.

## Implant design considerations

The truss cages are composed of a series of truss elements, referred to as truss cells, stacked in the *Z*-axis (Figures 1i–1j and 2k–2m). The basic truss cell is a 3D design element very common throughout architectural structures, and one that plays an integral role in modern construction techniques. It is a more sophisticated method, with a far better weight/strength ratio than simple columns and beams. They are analogous to the trusses employed in the Eiffel Tower, simultaneously light and open yet very strong. These individual truss cells are stacked on one another sequentially in the *Z*-axis, in greater numbers corresponding to the length of the void the truss cage is intended to occupy.

The shape of the implant must respect the anatomy and mimic the original configuration of bone (Figures 1h, 1n and 2k–2m). For long bone defects, juxta-articular implants have a metaphyseal flare (Figure 2m), and diaphyseal implants are more cylindrical (Figure 1i). Doing so limits the potential for soft tissue impingement, allows for optimal muscle function and maintains normal mechanical relationships within the overall limb. The shape of the truss design is paramount, as the shape of any patient-specific implant should in large part dictate the nature of any deformity correction that is achieved. The design of the implant must take into consideration a number of critical factors to ensure a favourable outcome. These include mechanical, anatomical and functional aspects that are unique to each case. Furthermore, the three-dimensional configuration of the implant must be designed to allow it to be successfully implanted through the planned surgical exposure, cognizant of the constraints imposed by local anatomy and prior implants.

Once the general size, shape and contours of the implant have been configured to closely match the original bone, one must consider how to stabilize the implant to allow osseointegration to occur while still allowing early active motion and protected weight bearing. This is planned in conjunction with the involved surgeon, who is aware of the possibilities as well as the specific plan for stabilization including IM nails, plates, screws and cerclage cables. The truss cage implant must often be designed with voids incorporated into the construct to accommodate the trajectory of screws that traverse the implant itself (Figures 1h, 1j, 1m, 1n and 2l, 2r–2t). Implants that are very near to a joint may ultimately replace the articular surface, at least in part. This may require a thin titanium “skin” to be incorporated on the exterior of the truss construct, that can be highly polished and will then better articulate with intact articular cartilage on the opposite side of the joint (Figures 2k–2m).

If there is a need to resect additional bone, or if the preoperative plan includes placing “blind” screws through the construct, it will often be useful to also design cutting jigs and drill guides to facilitate the actual procedure. When the current position of the region of interest is acceptably aligned, cutting jigs and drill guides can be configured and positioned based on the current implant or local anatomical features. If there is a plate already in place, the jigs and guides may be designed to rest in screw holes in the current implant when possible. The jigs and guides can be screwed to the current implant and will be very rigidly oriented with regard to the skeletal pathology. This will allow the jigs and guides to be fixed in space and will aid the surgeon to accurately cut or drill the bone. When the current position of the region of interest is not in acceptable alignment, these cutting jigs and drill guides should be configured and positioned based on local anatomical features, taking into consideration the planned change in position with respect to local anatomy. The possibilities are almost limitless when conducting virtual planning.

At this point it is time to consider definitive fixation of the bone/implant composite, with particular attention given to the use of either an IM nail (Figure 1) or a plate (Figure 2) as the means of providing immediate stability. IM nails are highly constraining devices and require the bones to be positioned and aligned correctly during both the virtual procedure and the actual procedure. Plates are inherently less constraining and allow greater latitude in terms of design considerations. Intra-medullary nails are mechanically advantageous in diaphyseal bone segments, but plates are better suited to metaphyseal or juxta-articular locations where a normal articulation needs to be preserved. Intramedullary nails are often used as the stabilizing element when the goal of reconstruction is an arthrodesis, including hind foot fusion nails to achieve an ankle arthrodesis or a long fusion nail to achieve a knee arthrodesis. Regardless of which type of adjunct fixation the surgeon prefers for a particular application, the design engineering team needs to consider the specific configuration of the remaining intact skeleton, the truss scaffold and the means used to provide stability of fixation to allow the composite construct to successfully incorporate.

To enhance rotational control, miniature spikes may be added to the struts on the end of some implants (Figure 1i). This provides not only torsional resistance, but also limits the potential for shear across the bone/implant interface. The titanium struts are inherently rough and resist torsion, but this can be enhanced significantly with either these small “cleats” or by designing macro elements into the implant that correspond to known bone defects. It is also possible to design intramedullary tapered truss extensions that can provide torsional stability once seated against the bone. By taking advantage of irregular skeletal geometry the inherent shear/rotational resistance at the bone/implant interface can again be significantly enhanced. The truss cages are currently produced with an Arcam printer (Mölndal, Sweden) using the “electron beam melting” (EBM) process. Using this method to print the titanium truss cage allows the surface characteristics to be refined. The struts themselves are manufactured with a rough surface, creating an implant that is highly biocompatible, encouraging eventual osseointegration between bone graft and implant (designed by 4WEB Medical, Frisco, Texas; manufactured by 3D Systems, Littleton, Colorado). These implants are covered by multiple US and international patents, and are Food and Drug Administration (FDA) approved on a compassionate basis as patient-specific custom devices.

If the design incorporates plates to augment stability, the junction of the plate and the truss becomes a stress concentration focal point, and the implant is subject to potential failure at this junction. Consideration should be given to a design where the plate element spans the entire truss component, to limit this potential for premature failure. A slotted hole in the plate is often advantageous, simultaneously permitting some motion for ease of insertion while also allowing compression to be applied after the truss cage is in place (Figures 2l, 2r–2t).

## Discussion

Custom designed patient-specific 3D printed titanium truss cages represent an innovative approach for the management of an extremely difficult and demanding clinical problem. This process of 3D modelling/printing is most valuable clinically when the pathology is most abnormal. Although currently this particular 3D modelling/printing treatment strategy is rarely used [35], preliminary results suggest it may eventually have a substantially greater role in challenging cases.

While analogous in some ways to using titanium spine cages to reconstruct segmental skeletal defects [61, 62], this approach differs in several important respects. By placing the implant into an induced membrane, the potential for rapid and complete osseointegration is maximized [62]. Additionally, through meticulous planning in a virtual procedure, these implants can be designed to precisely match the unique contours of the remaining host bone. These implants can thus create inherent mechanical stability, while also assisting in the correction of any existing deformities.

Post-traumatic deformities are not uncommon, and the complexity of the pathology generally reflects the severity of the initial injury. Reconstruction of the mangled extremity by any means is often long and arduous for both surgeon and patient, and frequently involves protracted periods of external fixation [63]. In many parts of the world, gradual correction through distraction osteogenesis using external fixation is an accepted standard of care. However, these methods can be associated with their own set of complications including pin site infections, non-unions, scarring and limitation of motion [64]. In wealthier economies there has been a transition towards acute correction of angular deformities, in combination with gradual lengthening using telescopic nails. Segmental bone loss further complicates attempts at reconstruction, and again external fixation is often used to achieve bone transport through distraction osteogenesis [65, 66]. Direct reconstruction of a segmental defect with bone graft is unlikely to be effective when the defect size is above a 6 cm threshold, due to graft resorption [67,68]. The Masquelet induced membrane technique is an attractive alternative in some circumstances, but the ideal parameters with respect to timing and bone graft have not yet been fully defined [55–60]. Even this method is no panacea, and defects exceeding 8 cm are more likely to have complications with this approach [69].

A number of recent publications have illustrated the efficacy of custom designed patient-specific 3D printed implants to address complex orthopaedic pathology [23, 25, 27, 28, 30, 31, 35, 51]. Because of the unique nature of each case, these papers generally appear in the form of case reports describing the benefits in each unique situation. Only after our collective experience grows will it become possible to accumulate enough data to document whether or not this is a cost-effective strategy. However, it does appear to have genuine promise in highly selected cases. Other surgeons have reported success with a similar approach for complex distal tibial injuries, and in cases of failed ankle arthroplasty [35]. Our primary indication at this time is for large (>8 cm) segmental defects in the distal femur, where the remaining articular surface fragment is small (<2 cm) but preserved, in compromised hosts/wounds.

There are limitations inherent in this approach, principally reflecting the sophisticated nature of the technology involved. The computers and software necessary are available almost everywhere, but access to experienced medical modellers and orthopaedic engineers would be far more restricted. The ability to 3D print custom titanium implants is at this time still cost-prohibitive in most healthcare systems globally. These devices are not biodegradable, and there are legitimate concerns if one became infected that would then require radical debridement to eradicate. Finally, these implants could create potential issues if a subsequent arthroplasty was later deemed necessary.

## Conclusions

Virtual surgical planning requires close collaboration between the orthopaedic surgeon, the orthopaedic engineering professional and the biomedical design engineer. Patient-specific custom 3D printed titanium truss cages, used in conjunction with the Masquelet technique, are a promising new treatment option for managing complex trauma patients with segmental bone loss. This approach appears to have advantages for cases of massive juxta-articular bone loss, when other biological techniques may not be possible. In our limited experience, the distal femur appears to be the ideal location to utilize this approach, particularly when the size of the defect exceeds 8 cm and the host or wound is relatively compromised.

## Conflict of interest

SB is a senior orthopaedic engineering professional for 4Web Medical, of Frisco, Texas, the manufacturer of the implants described in the paper. The other two authors (KT and VG) have declared no potential conflicts of interest with respect to the research, authorship and/or publication of this article.
